# VKORC1 mutations in rodent populations of a tropical city-state as an indicator of anticoagulant rodenticide resistance

**DOI:** 10.1038/s41598-022-08653-8

**Published:** 2022-03-16

**Authors:** Cliff Chua, Mahathir Humaidi, Erica Sena Neves, Diyar Mailepessov, Lee Ching Ng, Joel Aik

**Affiliations:** 1 Environmental Health Institute, National Environment Agency, 40 Scotts Road, Environment Building, #13-00, Singapore, 228231 Singapore; 2 Pre-Hospital and Emergency Research Centre, Duke-NUS Medical School, 8 College Road, Singapore, 169857 Singapore

**Keywords:** Invasive species, Evolutionary theory, DNA sequencing, Mutation

## Abstract

Anticoagulant rodenticides are commonly used in rodent control because they are economical and have great deployment versatility. However, rodents with Single Nucleotide Polymorphism (SNP) mutations within the *Vkorc1* gene are resistant to the effects of anticoagulant rodenticide use and this influences the effectiveness of control strategies that rely on such rodenticides. This study examined the prevalence of rat SNP mutations in Singapore to inform the effectiveness of anticoagulant rodenticide use. A total of 130 rat tail samples, comprising 83 *Rattus norvegicus* (63.8%) and 47 *Rattus rattus* complex (36.2%) were conveniently sampled from November 2016 to December 2019 from urban settings and sequenced at exon 3 of *Vkorc1*. Sequencing analysis revealed 4 synonymous and 1 non-synonymous mutations *in Rattus rattus* complex samples. A novel synonymous mutation of L108L was identified and not previously reported in other studies. Non-synonymous SNPs were not detected in the notable codons of 120, 128 and 139 in *R. norvegicus*, where these regions are internationally recognised to be associated with resistance from prior studies. Our findings suggest that the prevalence of anticoagulant rodenticide resistance in Singapore is low. Continued monitoring of rodenticide resistance is important for informing rodent control strategies aimed at reducing rodent-borne disease transmission.

## Introduction

Rodents are identified globally to be pests and vectors for a variety of diseases transmissible to humans. Leptospirosis is a common zoonotic disease worldwide, communicable to humans through direct excreta contact from infected mammals, but rodents are well-known reservoirs for it. Approximately 1.03 million cases are reported with 58,900 deaths annually mainly in tropical regions and is a prominent zoonotic pathogen of concern in poorer regions of South and Southeast Asia^1,2^. Hantavirus haemorrhagic fever with renal syndrome (HFRS) or Hantavirus pulmonary syndrome (HPS) is another rodent-borne pathogen which can be contracted via inhalation of excreta aerosols. HFRS is widespread in Asia and Europe with mortality rates of 12%, while HPS is predominant in the Americas region with high mortality rates of about 40%^3,4^. Rats also serve as amplifying hosts to diseases transmitted by arthropod vectors that are parasitic to rats. The Oriental Rat Flea (*Xenopsylla cheopis*) can harbour *Rickettsia typhi*, which is responsible for causing murine typhus. Based on international seroepidemiological studies, murine typhus has a morbidity rate of 3–36% and is endemic in coastal areas of temperate climates in America, Europe and Asia^5^. Apart from the disease burden they carry, rodents increase economic costs to society by causing damage to structures, consumer goods and food. It was estimated that United States incurred losses of about $19 billion dollars per year due to the destruction caused by rats^6^.

In order to curtail the spread of rodent-borne diseases, reducing the vector population is a key approach implemented across many countries. Physical methods such as cage traps, snap traps and glue boards are frequently used. Physical traps do not result in any chemicals that could leach into the environment, pose no risk of secondary poisoning to other animals, and unintended trapped wildlife can be subsequently released. The use of chemical control in food production premises could be risky due to its potential to contaminate food. Therefore, the use of physical methods can be useful in such environments to avoid compromising food safety. Moreover, carcasses of rats can also be easily located and disposed to avoid the smell of decay. However, these methods are labour intensive and not as cost-effective, especially in countries where labour costs are higher than rodenticides^7^. Due to the neophobic nature of rodents, trap success is usually low and declines significantly after one to two days of trapping^8^.

Alternatively, the use of rodenticides offers a much cheaper option with lesser man hours required for placement and maintenance^9^. These baits can come in various forms such as bait blocks or in powdered form and can be mixed with food-based materials to make it highly attractive to rats for consumption. It is commonly placed directly into burrows or suspended as hanging bait blocks in the sewer lines, where it may be more physically challenging to deploy traps due to space constrains. The use of bait stations helps to reduce non-target animal ingestion. In addition to rodenticide effectiveness in reducing rat population^10^, long-term preventive baiting can also help in keeping rodent populations low.

Over the years, inappropriate or excessive use of these rodenticides has resulted in resistance, which has been a prevailing issue in many countries for the past few decades^11–13^. It has been postulated that the emergence of rodenticide resistance is associated with the selection pressure exerted from rigorous use of anticoagulants^11,12,14^. Another possible reason for increasing prevalence of rodenticide resistance is that it is transferrable from parent to offspring^15,16^. This poses a challenge for vector control, as physical control may be the only alternative for reducing rodent populations. Apart from the high labour costs associated with recurrent visits to check traps, only a fraction of rodents are removed from the site of infestation, allowing rapid population recovery in weeks to months. As such, the main objective of reducing the vector for rodent-borne diseases may only be temporary. Ironically, culling may even increase the prevalence of certain zoonotic pathogens in rats, as physical intervention disrupts the social hierarchy of rats^17^.

Anticoagulant resistance in rodents occurs when rodents develop resistance to the detrimental effects of anticoagulants, thus resulting in its efficacy loss despite correct application^18^. The mechanism for anticoagulants is such that these agents bind specifically to the Vitamin K epoxide reductase complex subunit 1 (VKORC1) enzyme and inhibits its activity. This enzyme is encoded by *Vkorc1* located in chromosome 1 for rats, chromosome 7 for mice and chromosome 16 for humans^19,20^. VKORC1 plays a crucial role in the vitamin K cycle, where its function is to reduce vitamin K 2,3-epoxide to its reduced form, vitamin K hydroquinone. It is an essential precursor for another enzyme γ-glutamyl carboxylase, which is involved in catalysing post-translational modification of vitamin K dependent proteins required for the cessation of bleeding^11,21,22^. Resistance to rodenticide happens when mutations known as single nucleotide polymorphisms (SNPs) occur in *Vkorc1*. This could cause non-synonymous mutations that results in a different amino acid being incorporated into the VKORC1 enzyme during translation. Consequently, anticoagulants are unable to bind effectively to VKORC1 to exert its effect^22–24^. Previous in-vivo studies have identified that SNPs in codons 139, 128 and 120 of exon 3 confer resistance in rats^20,25,26^.

Extensive studies on the distribution and prevalence of *Vkorc1* mutations have been carried out mainly in the European regions^16,26–30^. The growth and geographical expansion of mutant rat populations may compel an increased reliance on physical population control methods, thus increasing the cost burden to health and pest control authorities. The use of anticoagulant rodenticides is common in Southeast Asia but few studies have examined if rodenticide resistance has reduced the effectiveness of anticoagulant rodenticide use. One particular study in Indonesia found resistant phenotypes among rats in oil palm plantations that were subjected to intensive anticoagulant baiting^31^.

This study aimed to: (a) identify molecular indicators or novel mutations of anticoagulant resistance in exon 3 of *Vkorc1* for both the Norway (*Rattus norvegicus*) and Black rats (*Rattus spp.*), and (b) investigate the prevalence and distribution of these SNPs in Singapore in order to inform the development and review of rodent control strategies.

## Materials and methods

### Study site

Singapore is located in Southeast Asia, at the southernmost region of Peninsular Malaysia. There are approximately 5.7 million residents living on this island with a size of 728.3 km^2^^32,33^, a country with very high population density. A large majority of the island is comprised of high-rise buildings, whereby about 80% of the citizens reside in public housing known as Housing & Development Board (HDB) flats. General wastes from household buildings are consolidated into bin chutes before transportation by external waste management companies to incineration plants for treatment. There were a total of 37,527 licensed food establishments in Singapore as at 2019^34^, an average of about 51.5/km^2^.

### Ethics approval

This study was part of the National Environment Agency’s existing integrated national programme of rodent surveillance and control. Our study did not involve any human subjects. The Environmental Health Institute of the National Environment Agency, Singapore (NEA) reviewed the protocols and gave study approval (TS217). All applicable national guidelines for the care and use of animals were followed. Rodents trapped in the study did not belong to endangered or protected species. This study is reported in accordance with ARRIVE guidelines.

### Rat tissue sampling

*R. norvegicus* and *Rattus spp.* samples were selected for this study as they were the two most common species of rats in Singapore. This study employed a cross-sectional study with *R. norvegicus* and *Rattus* spp*.* samples collected via opportunistic sampling from shopping malls, common residential areas, back alleys of food establishments and parks located across Singapore. The map included in this study was created using the open-source geographic information system, QGIS version 3.16.4-Hannover^35^. Samples comprised of tails and carcasses provided by pest control companies directly after their population control operations. All rats obtained were physically (and not chemically) killed. For whole carcasses received, the tails were separated from the body and used for DNA extraction. Samples delivered or collected were labelled with their address location along with the species through morphological identification. A total of 83 *R. norvegicus* and 47 *Rattus spp.* were tested from November 2016 to December 2020. A small portion of flesh and muscle of each tail sample (1 cm from proximal end) were carefully excised, while avoiding the outer most layer of skin. This was done is to avoid any cross-contamination from the sampling carried out by the pest control, as well as any residual soil and dirt that could hamper downstream extraction procedures. DNA for these tail samples were extracted with the Qiagen DNeasy Blood & Tissue Kit (Qiagen), following their standard protocol provided.

### PCR amplification of *Vkorc1* and sequencing procedures

Polymerase Chain Reaction was carried out in 20µL reactions to amplify exon 3 of *Vkorc1*. Specific primers that were designed in accordance to Grandemange et al. (2009) were used to flank exon 3. Primer sequence are as follows: Exon 3 Forward 5’-TTTCACCAGAAGCACCTGCTGCC-3’ and Exon 3 Reverse 5’-ACACTTGGGCAAGGCTCATGTG-3’, with an expected amplified fragment size of 354 base pairs^28^. The amplification was carried out using the Phusion Flash High-Fidelity PCR Master Mix (Thermo Scientific) with the following cycle conditions: Initial denaturation at 98 °C for 10 s, 35 cycles of denaturation at 98 °C for 5 s, annealing at 69 °C for 15 s and extension at 72 °C for 10 s, followed by a final extension of 72 °C for 1 min. Amplified fragments were subsequently confirmed by gel electrophoresis on 1.5% agarose gel with GelRed Nucleic Acid Gel Stain (Biotium). Amplified fragments were then purified by FavorPrep Gel/PCR Purification Kit (Favorgen Biotech Corp.) before sending to 1st Base Asia for DNA sequencing.

### Species confirmation

For species confirmation, 34 random *Rattus* spp. individuals were chosen and 2µL of extracted DNA was amplified using primers that are specific towards cytochrome c oxidase subunit 1 mitochondrial gene (COI). A 657-bp segment from the 5’ region of COI was amplified using primers cocktails C_VF1LFt1/ C_ VR1LRt1^36^.

PCR amplification was carried out using the Phusion Flash High-Fidelity PCR Master Mix (Thermo Scientific) with the following cycle conditions: Initial denaturation at 94 °C for 2 min, 5 cycles of denaturation at 94 °C for 30 s, annealing at 50 °C for 40 s and extension at 72 °C for 1 min, followed by 35 cycles of denaturation at 94 °C for 30 s, annealing at 55 °C for 40 s and extension at 72 °C for 1 min, and final extension of 72 °C for 10 min. Amplified PCR products were checked using gel electrophoresis. PCR reactions were than purified and sequenced. All phylogenetic analysis were conducted in Geneious v11.0.3 (https://www.geneious.com). The sequences were assembled, trimmed and compared with the sequences available in BLAST (Basic Local Alignment Search Tool) and BOLD (Barcode of Life Data). A phylogenetic tree was constructed using the generalized time-reversible nucleotide substitution model with gamma rate heterogeneity and 1000 bootstrap replicates. The phylogenetic tree was visualized and edited in FigTree v1.4.4 and only bootstrap values ≥ 50% were considered (see Supplementary Fig. S1 online).

32/34 (94.1%) of the samples yielded high quality genetic data which were used in the phylogenetic tree in Supplementary Fig. S1. Two samples had low quality data that were inadequate for inclusion. The phylogeny shows that all the 32 *Rattus *spp. in Singapore fall into a unique distinct clade, indicating these individuals could be part of a new local isolated lineage of the *Rattus rattus* complex. The Rattini tribe taxonomy is considered to be one of the most intricate group of mammals, and researchers have not agreed upon an ultimate nomenclature^37,38^. The authors here decided to follow other studies and call these individuals as part of the *R. rattus* complex^39,40^, until further analyses help to elucidate this groups’ taxonomy.

### Screening of single nucleotide polymorphisms

Sequencing files were visually analysed using DNASTAR Lasergene SeqMan Pro and any low-quality ends were edited. BioEdit Sequence Alignment Editor was then used for ClustalW multiple alignment to compare the sequenced *R. norvegicus* and *R. rattus* complex samples to a published wild-type reference sequence from GenBank^25,41,42^ (Accession No. AY423047). SNPs identified while using the alignment editor were traced back to each individual sample in SeqMan Pro to determine if the mutation was heterozygous or homozygous.

## Results

### Rodent population

**Figure 1:**
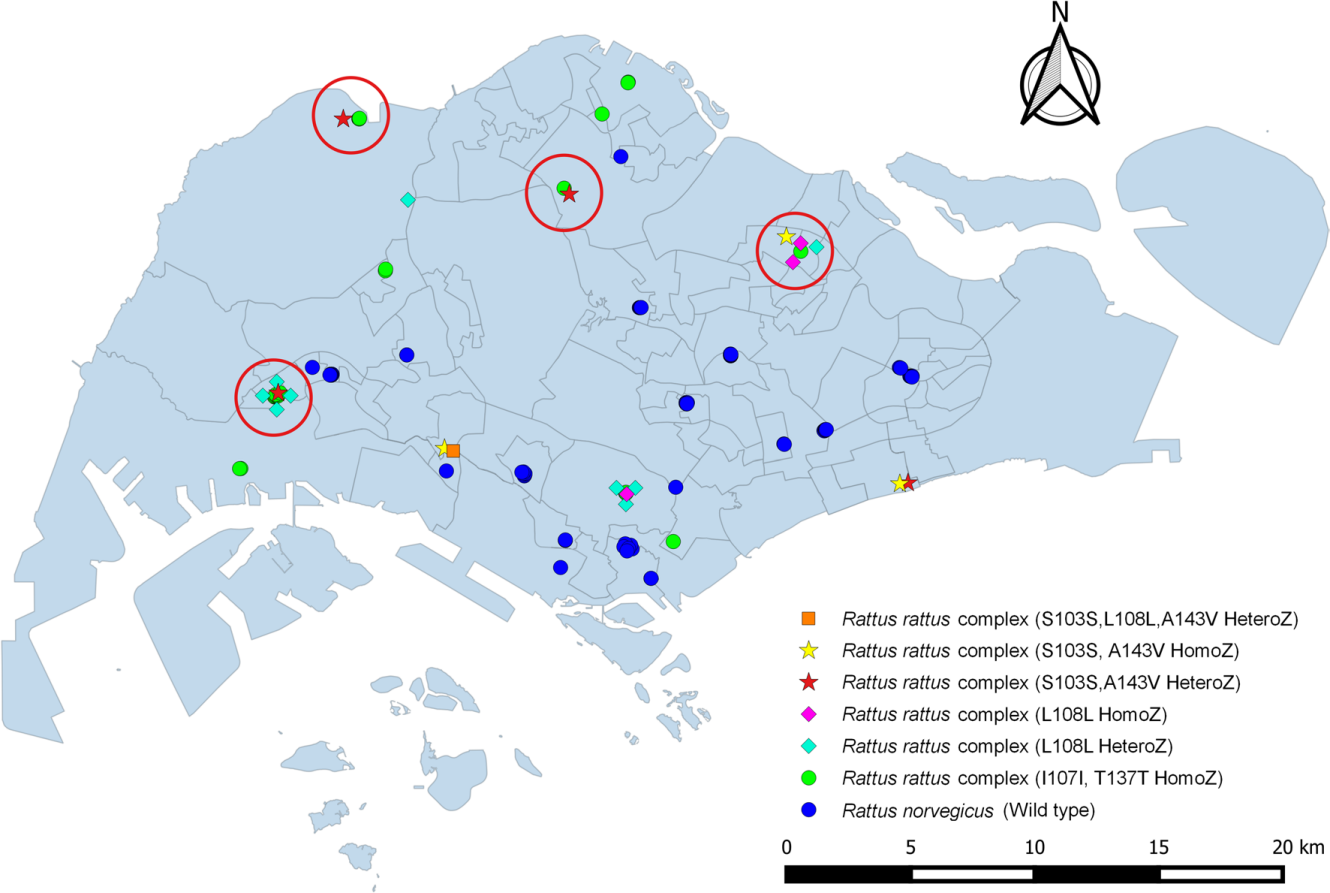
Map of Singapore depicting the locations of rats being sampled. Rats with the various SNPs are represented with different symbols and colours. Locations circled in red contains *R. rattus* complex samples with disparate types of mutations. Figure created with QGIS version 3.16.4-Hannover (https://qgis.org/downloads/).

*R. norvegicus* samples were collected from outdoor areas such as burrows and bin chutes around public residential estates. *R. rattus* complex samples were obtained from indoor premises within false ceilings of shopping malls, industrial buildings, private residential buildings and suburban regions like parks or nature reserves. The sampling locations are portrayed in Fig. 1.

In total, 83 of *R. norvegicus* and 47 of *R. rattus* complex tail samples were received and tested for SNPs. None of the *R. norvegicus* samples had mutations in codons 139, 128 or 120. No novel mutations were detected in the whole of exon 3, when compared with the GenBank AY423047 sequence.

**Figure 2:**
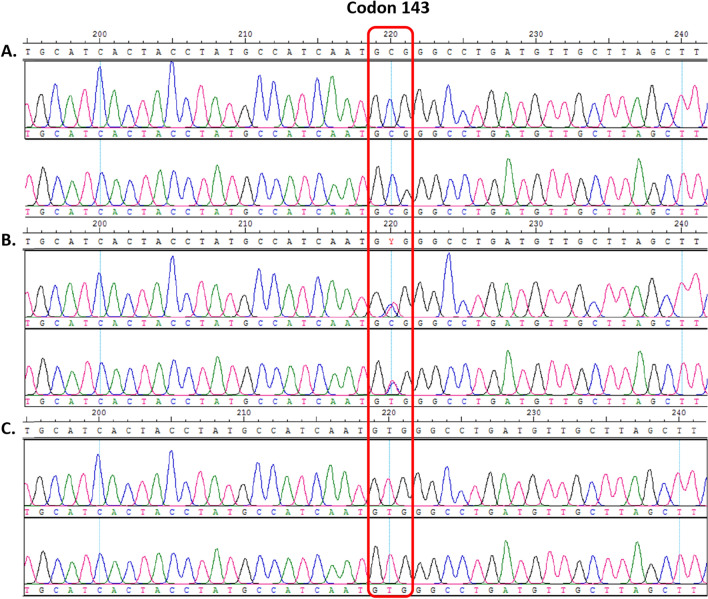
DNA nucleotide bases are each highlighted with a different colour. Electropherogram profiles of three different *Rattus rattus* complex samples depicting wild type **(A)**, heterozygous **(B)** and homozygous **(C)** SNP mutations at codon 143 of exon 3.

SNP mutations in *R. rattus* complex samples were not detected in the key codons of 139, 128 or 120. However, we detected the presence of heterozygous and homozygous mutations in other codons of exon 3. The electropherogram profiles in Fig. 2 shows the comparison of three different *R. rattus* complex samples at the same codon. Samples that have heterozygous haplotypes will have double-coloured peaks at a particular nucleotide location as seen in Fig. 2B. SNPs that were detected in exon 3 of *R. rattus* complex samples are denoted by the wild type amino acid, followed by the codon position and then the mutant amino acid. These SNPs and their codon location can be found in Table 1.

**Table 1:** Summary of SNPs found in exon 3 of *Vkorc1* with the respective substitution of amino acids of *R. rattus* complex (*n* = 47) and *R. norvegicus* (*n* = 83) when compared to the GenBank AY423047 wild type sequence.

					*Rattus rattus* complex			*Rattus norvegicus*		
Codon position	Codon & mutation	Codon WT	Codon mutant	Substitution mutation & amino acid change	Frequency (%)	Homozygous genotype	Heterozygous genotype	Frequency (%)	Homozygous genotype	Heterozygous genotype
103	S103S^a^	TCT	TCC	Ser103Ser	17.0	3	5	0	0	0
107	I107I^a^	ATC	ATA	Ile107Ile	100	47	0	0	0	0
108	L108L^a^	CTG	TTG	Leu108Leu	27.7	3	10	0	0	0
137	T137T^a^	ACC	ACT	Thr137Thr	100	47	0	0	0	0
143	A143V^b^	GCG	GTG	Ala143Val	17.0	3	5	0	0	0
No mutation observed					0	0	0	100	83	0

^a^SNP mutation that is silent.

^b^SNP mutation that brings about a substitution in amino acid.

*WT* wild-type sequence/amino acid.

### Results for SNPs in exon 3 of *R. rattus* complex

Five SNPs were detected at exon 3 for the *R. rattus* complex, of which one caused a substitutional mutation of GCG to GTG at codon 143 resulting in the amino acid valine replacing alanine (see Fig. 2). The other four mutations at codons 103, 107, 108 and 137 were silent. Allelic frequencies for both species of rats in exon 3 are summarised in Table 1.

All of the *R. rattus* complex samples carried silent homozygous SNP mutations in codons 107 and 137. In addition, 27.7% (13 out of 47, 3 homozygous and 10 heterozygous) of the samples had the silent SNP mutation in codon 108 (Leu108Leu). 17.0% of the samples (8 out of 47, 3 homozygous and 5 heterozygous) had SNP mutations in both codons 103 (Ser103Ser) and 143 (Ala143Val). These 8 samples, which were obtained from settings such as shopping malls and parks from 4 different locations and spaced 10 km or more apart across the country, displayed mutations that were disparate between the 4 locations (Fig. 1).

## Discussion

In this study, we sought to determine the prevalence of anticoagulant rodenticide resistance in Singapore to inform rodent management strategies. We did not find any evidence of anticoagulant rodenticide resistance in the *R. norvegicus* populations. Moreover, SNPS were absent for the whole of exon 3 for all Norway rat samples, identical to that of the wild-type. This finding was surprising, given that *R. norvegicus* was the most common species of rodents found in urban environments of Singapore^43^, and that anticoagulant rodenticide has been widely used by pest control operators (PCOs) to treat heavily rodent infested places in the last 20 years. The findings in our study for *R. norvegicus* differed from others undertaken in Netherlands, France, England, Germany, Hungary, Azores, USA and Argentina^12,14,15,28,30^. The majority of the missense SNPs proven to cause resistance in Norway rats that were detected in the European regions were Tyr139Cys, Tyr139Phe, Leu128Gln, Leu128Ser and Leu120Gln. Interestingly, *R. norvegicus* samples in Indonesia and Thailand (which are located in Southeast Asia) were reported to contain both missense and silent mutations^12^. A possible explanation for the absence of the above-mentioned SNPs in Singapore is that the urban conditions could be relatively harsh for Norway rats to thrive. In Singapore, there are well established refuse management and public cleaning programmes that reduces access to food waste and keeps the environment clean. Strict laws and regulations are in place to minimize littering, poor waste management and food hygiene practices in food retail establishments that can generate food sources to facilitate rodent population growth. This reduces the accessibility to food sources, leading to tougher competition amongst rat colonies. Even if spontaneous resistance mutations occurred within a few individuals of the rat population, they might not be fit enough to survive until they produce the next generation of pups. Similarly, a good and comprehensive rodent control programme that comprises of pulsed-baiting techniques^44^ and alternating between types of rodenticide usage and physical trapping strategy may also result in low resistance frequency.

In contrast, the *R. rattus* complex rats contained multiple SNPs within exon 3 itself comprising of a variety of SNPs that are either silent or missense mutations. Although SNPs were not detected in the widely studied codons of 139, 128 or 120 that have been proven to confer resistance^20,25,26^, it was interesting to find other mutations and this suggests at the possibility of local rat populations in acquiring rodenticide resistance phenotypes. In our study, *R. rattus* complex rats carried multiple silent mutations within exon 3 of *Vkorc1*, namely at codons Ser103Ser, Ile107Ile, Leu108Leu and Thr137Thr. This essentially means the nucleotide variants do not result in a substitution of amino acid, allowing the VKORC1 enzyme to retain its protein structure and function. The two most common silent mutations observed were Ile107Ile and Thr137Thr and this was found in all of the *R. rattus* complex rats carrying both SNPs in homozygous form. In fact, all of the *R. rattus* samples tested in New Zealand and India demonstrated the exact same pattern of silent mutation at both of these codons^41,45^. One reason for this finding could be the outcome of genetic bottleneck events or founder effect that occurred in the past. As SNPs in the *Vkorc1* gene is heritable from parents^15,16^, all that is required is for a single founder individual having these two SNPs to pass down the mutations to modern day rat populations. Due to Singapore’s strategic location as a trading hub since the nineteenth century, ship vessels frequent this island and it is likely that stowaway rats from neighbouring countries were imported many years ago. Nevertheless, it is interesting to note that *R. norvegicus* from Indonesia and Thailand have also been reported to exhibit similar SNP mutation patterns at codons 107 and 137^12^. Hence, the silent mutations in these two codons are not only exclusive to *R. rattus,* but also able to occur in *R. norvegicus* as well.

A new silent SNP mutation that has not been described before was identified in this study. It was located in codon 108 with the substitution of a single cytosine nucleotide to tyrosine. About a quarter of the *R. rattus* complex rat population possess the Leu108Leu variant, which is quite substantial given that there is no substitution of amino acid involved. The final silent mutation detected in this study was the Ser103Ser variant. What makes this SNP interesting is that all the *R. rattus* complex rats with it simultaneously also carried the Ala143Val SNP, the only amino acid substitution observed in this study. Rats that are heterozygous for the SNP at codon 103 were observed to be heterozygous for the Ala143Val variant and likewise if it was homozygous. It is quite likely that the Ala143Val genotype has a strong association with the 103Ser103 silent mutation in exon 3. The 4 identified locations that exhibited disparate genotypes amongst the *R. rattus* complex samples could suggest that they were unlikely from common lineages. If the 103Ser103 and Ala143Val genotype represents the initial emergence of resistance, then more extensive monitoring of the SNP mutation profile among rats in future is warranted. As this genotype is seen in the wild type VKORC1 protein of other species such as humans and mice, it is considered to be a neutral mutation^12^. However, a slightly more recent study in Indonesia in 2012 tested the Asian House Rats, *R. tanezumi*, sampled from areas with intensive coumatetralyl usage had comparable genotypic patterns. It was reported that the Ala143Val mutation was found in nine rats and seven of them displayed resistant phenotypes^31^. Further research is required to elucidate the mechanism by which this amino acid substitution truly confers resistance.

Molecular analysis of mitochondrial DNA from fossils found in present day traced that Norway rats historically originated from Southwestern China about 1.3 million years ago^46^. Also, for the Roof rats, a strong phylogeographic pattern indicates that they were native to South Asia and Indochina regions^47^. Despite studies depicting these two species having Asian origins, rodenticide resistance was first described in Europe and then United States about one to two decades after the introduction of rodenticide in the early 1950s^16,48,49^. This strongly suggests that the evolution of anticoagulant resistance could be the result of massive or improper use of warfarin in the western regions of the world where it was developed and used. As such, the exon 3 SNP profile of rats in Singapore was unexpected, since rodenticides have been used for the past 20 years. Likewise for China, anticoagulant rodenticides have been widely used for over 30 years and yet the frequency of resistance has been reported to be low^50^.

*Vkorc1* has been extensively studied and alterations to the protein structure is known to affect the blood clotting mechanism. Future studies can include investigating SNP mutation profiles of exon 1 and 2 for other non-synonymous mutations, coupled along with blood clotting response (BCR) test. It will also be interesting to investigate rodents’ ability in metabolising the ingested rodenticide by measuring gene expression of cytochrome P450. The P450 enzyme breaks down a wide range of drugs and an enhanced expression of certain P450 genes can increase drug metabolism rate^51^. This could result in anticoagulants being cleared from their cardiovascular system quickly, causing rodenticides to be ineffective.

Our results indicate that the prevalence of SNP mutations in the rat population in Singapore was low and it is reassuring that there is no widespread resistance. Nonetheless, further studies to provide a better geographical representation and spatial resolution of assessed rodenticide resistance would benefit the review of site-specific intervention strategies. Regular monitoring of the extent and evolution of mutations in rodent populations would facilitate the review of resistance management strategies.

Given that this was the first study carried out in Singapore since the registration and use of rodenticides, we did not have historical information on the status of anticoagulant resistance or types of SNP mutations for comparison over time. To the best of our knowledge, there were no recent studies that examined this either. We obtained our samples through convenience sampling and our results many not be generalizable to the entire population of rodents in Singapore. However, we have no reason to believe that rodenticide resistance in the areas from which the rodent samples were obtained had lower resistance compared to other areas that had no samples since population activity was higher and control activities more extensive in the former. While screening for SNPs in *Vkorc1* does provide some information on the mutations associated with resistance, it is a proxy of the actual level of resistance towards rodenticides.

## Conclusion

A new *Vkorc1* silent SNP mutation of Leu108Leu was discovered in a few *R. rattus* complex populations of this study, though it was not associated with anticoagulant resistance. Our study findings suggest that rodenticide resistance in Singapore was either low or absent, even after nearly two decades of rodenticide use. This provides support for the continued use of anticoagulants as a means for effective rodent population control. Continued rodenticide resistance monitoring will inform rodent control strategies for reducing rodent-borne disease transmission.

## Supplementary Information

Supplementary Figure S1.

## Data Availability

The data used in this study are owned by a third party. Requests for the data can be made to the Environmental Health Institute of the National Environment Agency at Contact_NEA@nea.gov.sg.
