# Supplementary material for: VKORC1 mutations in rodent populations of a tropical city-state as an indicator of anticoagulant rodenticide resistance
Source: Sci Rep. 2022 Mar 16;12:4553. doi: 10.1038/s41598-022-08653-8 (PMC8927331; doi:10.1038/s41598-022-08653-8)
Supplement: Supplementary file 1 — Supplementary Figure S1. [file 41598_2022_8653_MOESM1_ESM.docx]

**Supplementary Information**

**Single Nucleotide Polymorphisms (SNP) within *Vkorc1* in rodent populations of a tropical city-state: Implications for anticoagulant rodenticide use for rodent control**

Cliff Chua^1,*^, Mahathir Humaidi^1^, Erica Sena Neves^1^, Diyar Mailepessov^1^, Ng Lee Ching^1^, and Joel Aik^1,2^


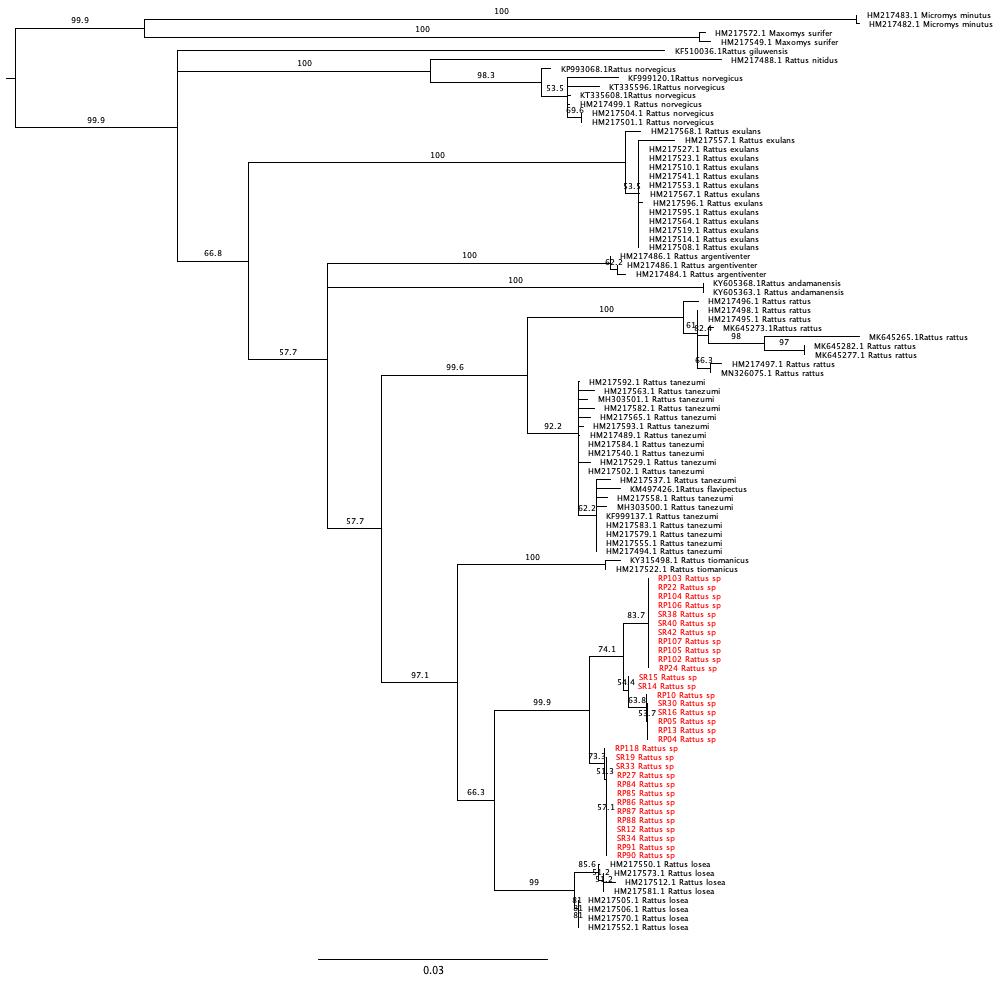
**Supplementary Figure S1.** Neighbor-joining phylogeny tree based on the COI gene. The scale bar indicates nucleotide substitutions per site and bootstrap values ≥50% are shown. The *Rattus spp*. sequences in this study are shown in red.
